# Functional Analysis of *OsCIPK17* in Rice Grain Filling

**DOI:** 10.3389/fpls.2021.808312

**Published:** 2022-01-25

**Authors:** Cong Gao, Xiuru Zhu, Shuai Lu, Jingbiao Xu, Rong Zhou, Jianying Lv, Yaoyu Chen, Yunying Cao

**Affiliations:** School of Life Sciences, Nantong University, Nantong, China

**Keywords:** rice, transcriptome, photosynthesis, grain filling rate, grain weight, starch and sucrose

## Abstract

We used mutant *cipk17* and Nipponbare in field experiments to analyze agronomic traits, photosynthetic parameters, transcriptome, and gene expression. The results demonstrated cytoplasmic localization of *OsCIPK17*, while GUS allogeneic (*A. thaliana*) tissue-staining and quantitative analysis showed the gene was expressed in many organs, including flower buds; furthermore, it was involved in root, stem, and leaf growth. Compared to Nipponbare plants, grain filling rate and final grain weight decreased in plants of the knockout mutant owing to a delay in attainment of maximum grain filling rate. Photosystem II (PSII) efficiency was also reduced. Enrichment analysis showed that the functions of differentially expressed genes (DEGs) focused on nucleoside-, nucleotide-, and lipid-binding, as well as hydrolase, transferase, and phosphorylase activities. Signaling pathways mainly included starch and sucrose metabolism, as well as photosynthesis. Additionally, some DEGs were verified by fluorescence analysis. The results showed that knockout of *OsCIPK17* affected photosynthesis and starch-, sucrose-, and amino acid metabolism-related gene expression; furthermore, the mutation reduced PSII utilization efficiency, it blocked the synthesis and metabolism of starch and sucrose, and affected the formation and transport of assimilates, thereby reducing final grain weight.

## Introduction

Signal transduction in plant cells begins with the perception of external signals, such as different environmental stimuli that are recognized by specific receptors and transmitted to a second messenger in the cell, which in turn triggers a downstream cascade reaction that ultimately results in specific physiological and biochemical changes leading to an overall biological effect (Zhu, 2001). Calcium ions (Ca^2+^) are second messengers widely involved in regulating plant growth and development (Heple, 2005). Further, Ca^2+^ receptors can be structurally and functionally divided into two categories. The first comprises the sensor-relay receptor type containing the Ca^2+^ binding function, which has no kinase activity and can regulate the expression activity of downstream genes by interacting with proteins such as calmodulin (CAM), calmodulin-like protein (CML), and calcineurin B-like protein (CBL), all of which can modulate other effector proteins to trigger downstream responses that transmit signals. The second category of Ca^2+^ receptors comprises signal sensor responders that can combine with Ca^2+^ to act as a kinase, such as the calcium-dependent protein kinase (CDPK), which combines with Ca^2+^ to self-induce a required conformational change (Kudla et al., 2010; Boudsocq and Sheen, 2013).

The CBL protein belongs to the serine/threonine protein phosphatases, a peculiar category of calcium-binding proteins in plants. The CBL protein has a typical Ca^2 +^-binding domain, namely the EF-hand domain, which, during Ca^2+^-mediated signal transduction, must interact with Ca^2+^-dependent serine/threonine protein kinase (CIPK) to transmit the signal to the cell (Luan et al., 2002; Xiang et al., 2007). CIPK is a CBL-dependent interacting protein kinase and its sequence is relatively conserved. All CIPK proteins contain an N-terminal kinase domain and a C-terminal regulatory domain, in which the C-terminal contains an NAF domain that specifically binds to CBL (Albrecht et al., 2001; Lee et al., 2007). CIPK is a large family whose number of members varies among species. Thus, crop plants such as corn have the largest number of CIPK genes with up to 43 (Chen et al., 2011b), while green beans and peas have only five and one, respectively (Mahajan et al., 2006). The CIPK family was first reported for *Arabidopsis thaliana*, with 26 members (Liu and Zhu, 1998). Similarly, rice has many CIPK gene family members; 33 have been identified to date and 15 have been cloned (Xiang et al., 2007; Chen et al., 2011a). Studies have shown that CIPK is involved in plant growth. For example, in rice CIPK31 is involved in seed germination and seedling growth under abiotic stress (Piao et al., 2010), and in millet, gene expression of *CIPK6* and *CIPK16* changes with growth stage under drought stress (Yu et al., 2016). Nonetheless, the function of *CIPK17* in plants, especially in rice, has not yet been explored.

In rice, grain filling is closely related to grain weight and to the position of the grain on the panicle. Generally speaking, superior grains in the middle and upper positions on the panicle have an advantage over inferior grains at lower positions during grain filling (Yang, 2010). Compared with superior grains, the grain filling process in inferior grains in the lower part of the panicle affects grain yield and quality more significantly (Cao et al., 2016; Li et al., 2021). The asynchronous filling of the superior and inferior grains on the panicle may be closely related to sucrose transformation and starch synthesis (Zhao et al., 2021). Some researchers have found that inferior-grain filling is easily affected by the environment, whereas superior-grain filling is affected by heredity to a greater extent (Zuo et al., 2002). To date, many researchers have focused the molecular mechanism underlying inferior-grain filling (Zhang et al., 2015, 2019), while much less research has been conducted on superior-grain filling. Mutants are commonly used in gene function research (Hu et al., 2021). In this study, we constructed a mutant line of *OsCIPK17*. Preliminary studies have shown that this gene is involved in grain development in rice. The purpose of this study was to reveal the functional role of *OsCIPK17* in rice grain filling and provide a theoretical basis for further cultivation of rice varieties.

## Materials and Methods

### Planting, Identification, and Sampling of Materials

The experiment was conducted in the botanical garden of Nantong University (32°1′N, 120°53′E). The materials used included parent Nipponbare (NIP), and the mutant line 41 (*cipk17*) obtained by *OsCIPK17* knockout using the CRISPR-Cas9 method. In 2018, mutant *cipk17* and parent NIP were field planted and leaf DNA was extracted. Positive seedlings were identified by cloning sequencing and primer amplification at the target with specific primers according to Hua et al. (2017). T1 and T2 generations were planted in 2019 and 2020, respectively. They were sown from May 17 to 19 and transplanted in the field from June 14 to 16, with plant and row spacing of 25 cm, at 1 seedling per hill. Before transplant, 225 kg urea and 275 kg compound fertilizer were applied per hectare; then, 225 kg urea and 300 kg compound fertilizer were applied at tillering, and 300 kg compound fertilizer was applied at panicle initiation. Water-layer height was kept at 1–2 cm during the entire growth cycle. All other management practices were as per conventional high-yield cultivation in the region.

### Subcellular Localization and GUS Analysis

The full-length CDS of *OsCIPK17* was amplified using gene-specific primers and cloned into the pCAMBIA-2300-35S-N-eGFP-OCS vector (p2300-eGFP) to generate N-terminal eGFP fusion proteins (eGFP-OsCIPK17) using the ClonExpress^®^ II One Step Cloning Kit (Vazyme). All constructed vectors were transformed into *Agrobacterium* strain GV3101. Positive GV3101 strains carrying the above vectors were transiently transformed into the leaves of 4-week-old tobacco plants (*Nicotiana benthamiana*). Laser confocal microscopy (Leica TCS SP8) was used to observe gene localization in the lower epidermis of tobacco plants.

The promoter fragment of *OsCIPK17* was amplified using the gateway method with gene-specific primers (Supplementary Table 1). pENTR/D-Topo vectors were used to construct the entry vector. The LR reaction was ligated to pMDC163 to obtain the GUS fusion protein (GUS-OsCIPK17). Heterologous *A. thaliana* positive transgenic plants were obtained after hygromycin screening. The expression of *OsCIPK17* in seeds, leaves, flowers, pods, and other tissues was detected by GUS tissue-staining and activity analysis using the fluorescence method.

### Analysis of Grain Filling Characteristics and Determination of Photosystem II Utilization Efficiency and Starch Content

Approximately 200 panicles that headed on the same day were selected and tagged as representative of the T2 generation of each genotype. The flowering date and position were recorded for each spikelet on the tagged panicles. Twenty to 30 tagged panicles from each material were selected every 6 days from anthesis to maturity; superior grains were sampled according to Cao et al. (2016). The used superior grains include all grains except the second grain of the primary branch in the upper third of the panicle. One part was rapidly frozen in liquid nitrogen and stored at −80°C for RNA-Seq and gene expression analysis. The remaining part of the sampled panicles was placed in an oven at 105°C for 30 min and dried to a constant weight at 80°C for 72 h to measure grain dry mass and perform grain-filling analysis as described by Cao et al. (2016).

The photosynthetic parameter photosystem II (PSII) use-efficiency (Phi2) was determined at fully tillering stage (FTS, July 18–20), heading and flowering stage (HFS, August 10–12), and middle milky stages (MMS, September 3–5) in parent NIP and in the mutant *cipk17* using Photosynq MultispeQ V2.0 (United States). Each material was tested six times. The starch content was determined by anthrone colorimetry (Wang et al., 2016).

### RNA-Seq Analysis and Protein-Interaction Network Construction

Owing to the significant difference in the 24-DAF (days after flowering) superior grains weight of mutant cipk17 and NIP, the 24-DAF superior grains were collected and sent to Guangzhou Yongji Biotechnology Co., Ltd., for mRNA transcriptome sequencing with three replicates per sample. The sequencing platform used was an Illumina NovaSeq 6000.

Plant ontology (PO) and gene ontology (GO) enrichment analyses were performed using the online website PlantGSEA. Kyoto Encyclopedia of Genes and Genomes (KEGG) enrichment analysis was performed using the GSEA V4.1 software with default settings. Genes with significantly different expression levels were imported into the online website String to obtain the protein–protein interactions. Then, Cytoscape V3.8.2 was used to construct the interaction network and further screen sub-modules and hub-genes.

### Differential Gene Expression

Total RNA of NIP and *cipk17* was extracted according to manufacturer instructions of the Total RNA Kit (TIANGEN). First-strand cDNA was obtained using the ReverAid First Strand cDNA Synthesis Kit (Thermo). Differentially expressed genes (DEGs) were determined by RT-qPCR using FS Universal SYBR Green Master (Rox) according to Cao et al. (2013) for three replicates with specific primers (Supplementary Table 2), and actin-1 (LOC_Os03g50885) as an internal control. All RNAs were extracted from the corresponding grains.

### Data Analysis

All data were collected from at least three technical and biological replicates and expressed as means ± SD. Statistical analyses were performed using SPSS V19.0. Significant differences were determined using Duncan’s test and graphs were drawn using the OriginPro V2021b.

## Results

### Identification of Mutants

**FIGURE 1:**
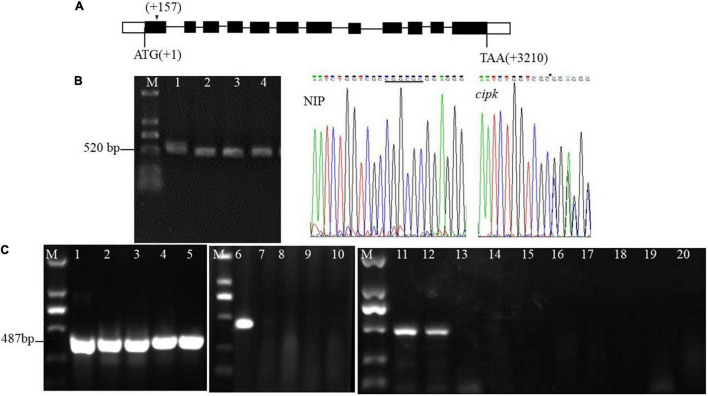
Target design of *cipk17* and identification of mutant lines. **(A)** *OsCIPK17* gene structure and target location; **(B)** PCR amplification of the target fragment of cloning and sequencing method and sequencing results of the target sequence. M: DL2000 maker; 1–4: 4 individual plants of mutant *cipk17*; Nip: it is the sequencing map at the target of wild-type Nipponbare; *cipk17*: sequencing map at target (AATCTGGTCGCCGGCGCGGAGGG) of mutant. The underline shows that there is six bases CGGCGC in wild-type, while are missing in the mutant line at the arrow; **(C)** the mutant *cipk17* was identified by Hua et al. (2017). 1–5: screening the annealing temperature Tm of specific primers of the *OsCIPK17* gene by gradient PCR with the method of primer design at the target. Tm is 68, 69, 70, 71, and 72°C, respectively; 6–10: identification of wild-type and mutant *cipk17* with specific Tm (72°C), 6 is Nipponbare, and 7–10 is different individual plants of mutant *cipk17*; 11–20 are the identification of tillers in the same single plant of mutant. 11–12 are Nipponbare, 13–20 are different tillers of the same plant in mutant line, respectively; M: DL2000 maker.

The target of the mutants in this study (AATCTGGTCGCCGGCGCGGAGGG) is on the first exon (157–174 bp downstream of ATG) (Figure 1A). The mutant lines were sequenced by TA cloning, and the results showed that the mutation caused a 6 bp deletion (CGGCGC) at the 12th bp of the target (Figure 1B). Use of the method of Hua et al. (2017) further confirmed that different individual plants of the mutant and multiple tillers in the same single plant were all mutants (Figure 1C).

### Subcellular Localization and GUS Analysis of *OsCIPK17*

**FIGURE 2:**
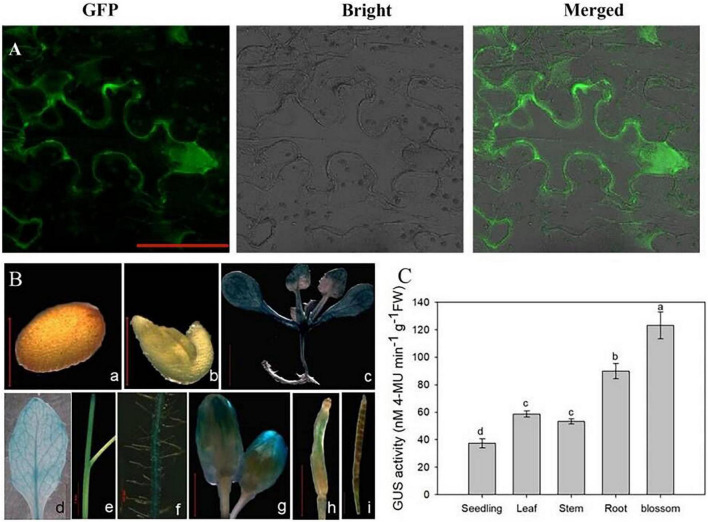
Subcellular localization, tissue expression, and quantitative analysis of *OsCIPK17*. **(A)** Subcellular localization of *OsCIPK17*. **(B)** The expression of *OsCIPK17* by GUS staining. **(C)** Quantitative analysis of GUS activity of *OsCIPK17* in *Arabidopsis* tissue by fluorescence method. Different lowercase letters above the bar of panel **(C)** indicate that there were significant differences among the tissues at *p* < 0.05.

Understanding the subcellular localization of genes is helpful for clarifying the expression pattern and elucidating gene functions. Subcellular localization analysis showed that the eGFP-OsCIPK17 fusion protein was significantly expressed in the cytoplasm of tobacco epidermal cells (Figure 2A), indicating that CBL in the cytoplasm is likely to combine with OsCIPK17 to form a CBL–CIPK complex and further regulate the expression of downstream genes because the cytoplasmic location of the complex is mainly determined by CBL (Ma et al., 2020).

To further understand the tissue expression pattern of *OsCIPK17*, the GUS-OsCIPK17 plasmid was transferred into *A. thaliana* and expression of the gene was determined by GUS tissue-staining (Figure 2B). The results showed that *OsCIPK17* was strongly expressed in 2-week-old seedlings and in the maturation zone of the root (Figures 2B-c/f), but not in the seed coat or the embryo (Figures 2B-a/b). *OsCIPK17* was detected in the rosette leaves and stems of *A. thaliana* grown in the soil for 35 days (Figures 2B-d/e). *OsCIPK17* expression in flower buds was high at flowering (Figures 2B-g) but became weaker during pod development (Figures 2B-h/i). To further verify the results of GUS tissue-staining, quantitative detection of GUS activity was analyzed in transgenic *A. thaliana* using the fluorescence method (Figure 2C). The results showed that GUS activity of the *OsCIPK17* gene was highest in flower buds, approximately 3.3 times that recorded for seedlings. Meanwhile, the corresponding activity in the root was approximately 2.4 times that observed for seedlings. Finally, in leaves and stems, the activity was 1.6 and 1.4 times that of seedlings, respectively.

### Effects of *OsCIPK17* Knockout on Superior Grains Filling, Photosystem II Utilization Efficiency and Starch Content

**FIGURE 3:**
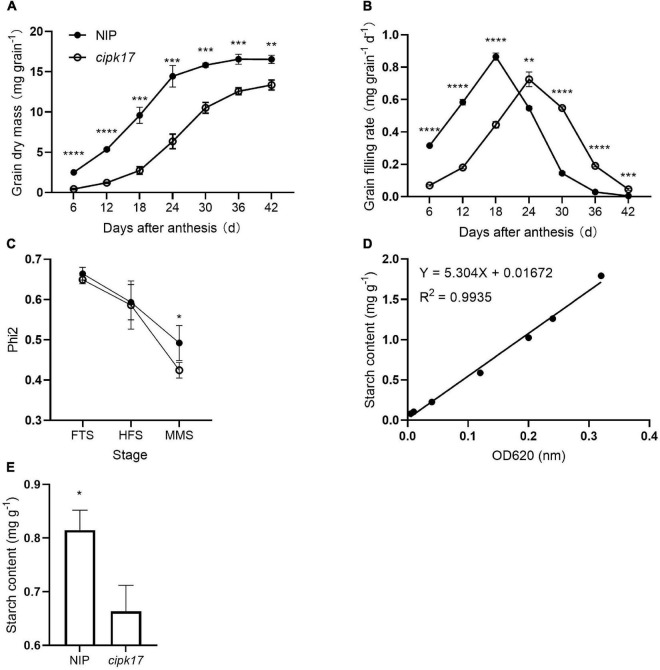
OsCIPK17 can affect grain filling process and starch accumulation. **(A)** Grain-weight increasing dynamics curves of superior grains; **(B)** grain-filling rate curves of superior grains; **(C)** changes of PSII utilization efficiency (Phi2) of rice flag leaves at different growth stages. FTS, fully tillering stage; HFS, heading and flowering stage; LHS, the later heading stage. Phi2: PSII utilization efficiency. **(D)** Linear regression equation for starch content. **(E)** Determination of starch content in rice grains at mature. ****p < 0.0001, ***p < 0.001, **p < 0.01, and *p < 0.05.

Figure 3 shows the change in superior grains weight, grain filling curves and starch content of NIP and *cipk17*. Clearly, grain weight in NIP plants was higher than that in the mutants throughout the filling period (Figure 3A). Before reaching a maximum filling rate (G_max_), the grain filling rate in NIP panicles always exceeded that of the mutant panicles. However, when the maximum filling rate was reached, the rate was higher in grains of the mutant than in those of NIP plants. The time (T_max_) to reach G_max_ was greater in the mutant *cipk17* than in the NIP parent. These results indicate that knockout of *OsCIPK17* delayed grain filling and reduced grain weight. As shown in Table 1, the mean grain weight in NIP was higher than that in mutant *cipk17*, and the grain weight of mutants decreased by 23.7%. The mutant reached G_max_ approximately 7.2 days later than NIP. Consistently, G_max_, mean filling rate (G_mean_), and the active filling period of NIP were all greater than those that characterized of the mutant.

**TABLE 1:** Characteristic parameters of superior grain-filling.

	Grain weight (g)	T_max_ (d)	G_max_ (mg grain^–1^ d^–1^)	G_mean_ (mg grain^–1^ d^–1^)	D (d)
NIP	21.25	18.27	0.86	0.55	29.86
*cipk17*	16.22	25.44	0.73	0.48	27.87

*T_max_, the time reaching a maximum grain filling rate; G_max_, the maximum grain-filling rate; G_mean_, the mean grain-filling rate; D, active grain-filling period; d, day.*

In addition, the utilization efficiency of PSII in the flag leaf at different stages of rice growth was measured (Figure 3C). The results showed that Phi2 of the mutant was lower than that of NIP at the FTS, HFS, and MMS. The difference between NIP and *cipk17* was significant in the MMS. These results indicated that the photosynthetic efficiency of the mutant was lower than that of NIP, and that the difference in photosynthetic efficiency may be the main reason for the differences in grain filling between the two materials tested. Finally, we measured the content of starch, a photosynthate. As can be seen from the figure, the starch content of the mutant was significantly lower than that of NIP (Figures 3D,E).

### Quality Control of Transcriptome Data and Screening of Differentially Expressed Genes

**FIGURE 4:**
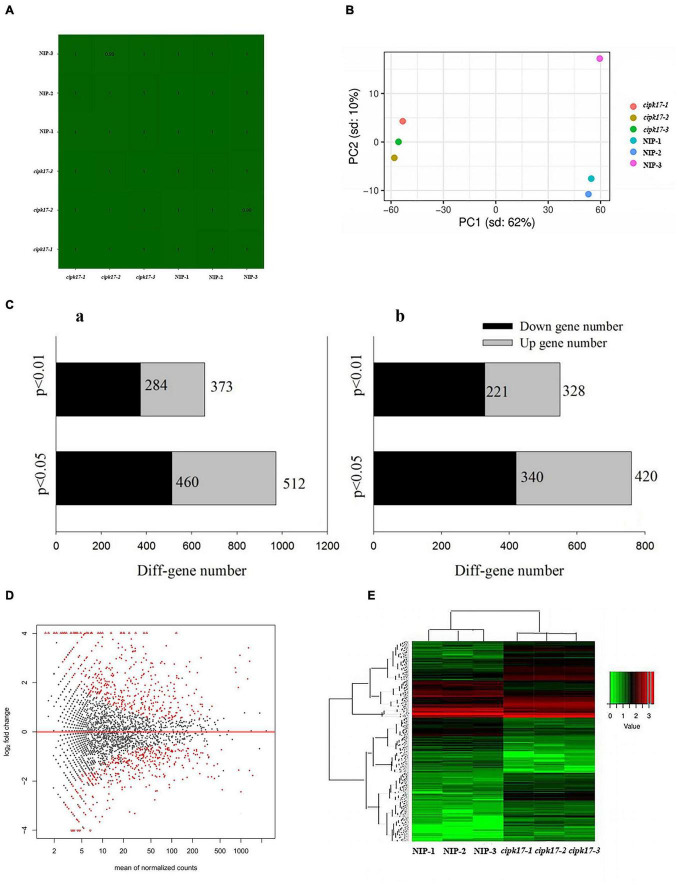
Quality control of transcriptome data and expression analysis of differential genes. **(A)** Correlation cluster analysis of samples. **(B)** Principal component cluster analysis of sequencing samples. **(C)** Quantitative analysis of differential genes screened by transcriptome sequencing; a of panel **(C):** differential genes screened by *p*-value; b of panel **(C):** differential genes screened by fold change threshold. **(D)** Volcano map of differentially expressed genes. The *X*-axis is the average expression value of all samples used for comparison after standardization, and the *Y*-axis is log_2_foldchange. The difference genes are marked in red with *p*-value < 0.05. **(E)** Cluster heat map of differentially expressed genes. Red indicates high expression genes and green indicates low expression genes.

To clarify the molecular mechanism by which *OsCIPK17* affects grain filling, the 24-DAF superior grains of mutant *cipk17* and NIP were selected for transcriptome sequencing. First, the sequencing data quality was analyzed. It is clear from the sample correlation cluster analysis (Figure 4A) that the correlation coefficient of each sample reached more than 0.99. Principal component cluster analysis (Figure 4B) showed that the NIP and mutant *cipk17* clustered together. PC1 contributed 62% of the difference between the NIP and mutant *cipk17*, and PC2 contributed 10%. The two results jointly prove the reliability of the data and meet the requirements of the subsequent analysis.

The detected genes were further analyzed and the differences between the two materials were reflected in a differential volcanic map (MA) (Figure 4D). Of the 41,757 genes detected, 2963 were found to be DEGs, including 1411 upregulated genes and 1552 downregulated genes. After preliminary *p*-value screening, 972 genes, including 512 upregulated genes and 460 downregulated genes, were significantly differentially expressed (*p* < 0.05). Among them, 657 genes were significantly different (*p* < 0.01), including 373 upregulated genes and 284 downregulated genes. Furthermore, despite the absence of any significant difference in expression between NIP and *cipk17*, the *p*-value was significant. Fold Change ≥ 2 was considered up-regulated, while Fold Change ≤ 0.5 was considered down-regulated. Through the fold change threshold screening, we found 760 genes with significant differences (*p* < 0.05), of which 420 were upregulated and 340 were downregulated. Additionally, 549 genes were screened that showed highly significant differences (*p* < 0.01) in expression; among these, 328 were upregulated and 221 were downregulated (Figure 4C). Concomitantly, heat maps of DEGs were drawn to observe the expression in different samples (Figure 4E).

**FIGURE 5:**
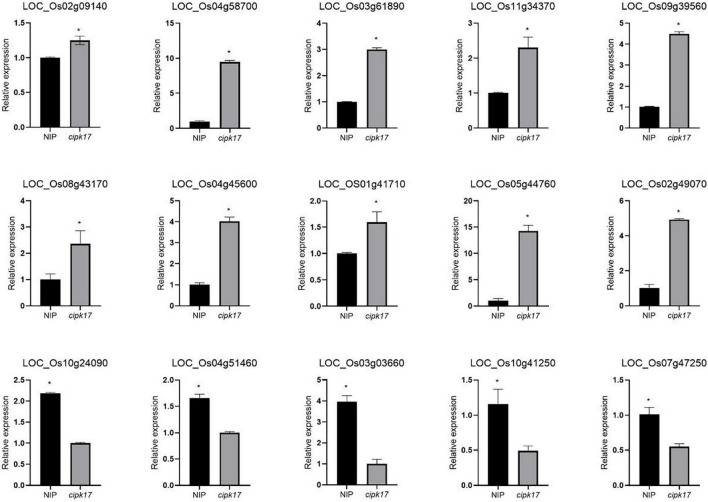
Fluorescence quantitative expression analysis of differential genes. Asterisks above the bar indicate that there were significant differences between NIP and *cipk17* material at *p* < 0.05.

Finally, 15 DEGs related to the synthesis and transportation of starch and sucrose were selected for RT-qPCR (Figure 5). The variation trend of DEGs in NIP and mutant *cipk17* was consistent with the results of transcriptome sequencing, which proved the reliability of transcriptome data. The results showed that *OsCIPK17* regulated grain development in rice.

### Enrichment Analysis of Differentially Expressed Genes

**FIGURE 6:**
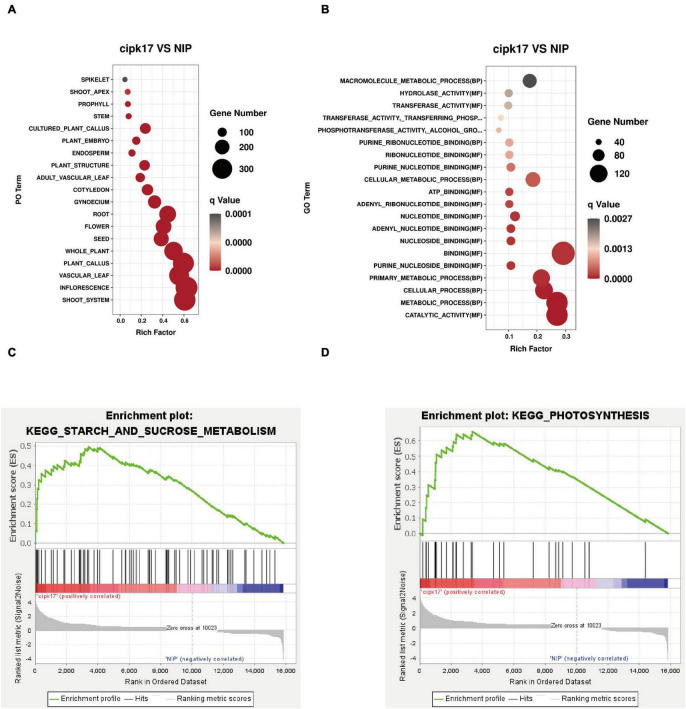
Enrichment analysis of differentially expressed genes. **(A)** PO enrichment analysis of differentially expressed genes; **(B)** GO enrichment analysis of differentially expressed genes; **(C)** GSEA enrichment analysis of starch and sucrose metabolism in KEGG terms; **(D)** GSEA enrichment analysis of photosynthesis of KEGG terms.

The results of PO enrichment analysis are shown in Figure 6A. DEGs were enriched in rice roots, stems, and leaves, indicating that *OsCIPK17* may be widely involved in rice growth. Furthermore, differential genes were enriched in reproductive organs, such as flowers and seeds. Through further subdivision, the differential genes were enriched in pistil and endosperm, indicating that *OsCIPK17* plays a vital role in regulating the development of rice grains.

In addition, GO enrichment analysis was conducted to clarify the functions of DEGs (Figure 6B). As shown in figure, differential genes were enriched in 42 functional aspects. Interestingly, differential genes were not enriched in the cellular component (CC) category, indicating that *OsCIPK17* had limited influence on cell composition. The functions of differential genes mainly focused on biological processes (BPs) and molecular functions (MFs). The first 20 functions were drawn into bubble plots according to the degree of enrichment significance. In the MF category, differential genes were mainly enriched in binding and enzyme activity. The binding effects mainly include nucleosides, nucleotide and lipid binding. The enrichment of many terms of nucleoside/nucleotide binding indicated that *OsCIPK17* may be widely involved in the fundamental growth and development processes of rice, such as ATP and nucleotide binding. ATP is widely involved in energy metabolism as an energy carrier. Nucleotides are mainly involved in the synthesis of nucleic acids, an essential component of all known forms of life, and the most essential substance of all biomolecules. Enzyme activities mainly include hydrolases, transferases, and phosphorylases. The enrichment of many hydrolases and transferases indicated that *OsCIPK17* may play an important role in the degradation and transfer of substances in rice. A large number of phosphorylases indicated that *OsCIPK17* may act as a kinase in protein phosphorylation and modification in rice. Phosphorylation is one of the most important covalent modification modes in organisms and is part of nearly all basic life activities. These results show that *OsCIPK17* is widely involved in basic growth and developmental processes in rice.

The KEGG was used for gene set enrichment analysis (GSEA), which revealed two important gene sets, namely, starch and sucrose metabolism, and photosynthesis, that were over represented (Figures 6C,D). Fifteen terms were significantly enriched whose functions mainly focused on photosynthesis, metabolism of its products, and amino acid metabolism (Table 2). Photosynthesis and photosynthate metabolism include photosynthetic antenna-protein metabolism, starch and sucrose metabolism, carbon fixation, and fructose and mannose metabolism. The antenna protein, a light-trapping protein, is a component of PSII. In turn, starch and sucrose are the two main end-products of photosynthesis. Sucrose is the main form of transfer of photosynthate from the leaves to sink organs, while starch is a storage form of carbon. These findings suggest that knockout of *OsCIPK17* may affect the leaf photosynthetic capacity for light harvesting, thus negatively affecting photosynthesis. The end products of photosynthesis, i.e., starch, and sucrose, affect the metabolism of fructose and mannose. Finally, *OsCIPK17* knockout affected plant phenotype.

**TABLE 2:** Kyoto Encyclopedia of Genes and Genomes enrichment analysis of transcriptome data.

NAME	ES	NES	NOM *p*-value
PHOTOSYNTHESIS	0.65950525	1.8786155	0
PHOTOSYNTHESIS-ANTENNA_PROTEINS	0.71647537	1.7724456	0.002392344
BUTANOATE_METABOLISM	0.7015285	1.7625161	0.004756243
STARCH_AND_SUCROSE_METABOLISM	0.49470386	1.5635237	0.004065041
SYNTHESIS_AND_DEGRADATION_OF_KETONE_BODIES	0.80672485	1.5449811	0.02052786
CARBON_FIXATION_IN_PHOTOSYNTHETIC_ORGANISMS	0.48197404	1.4935884	0.015368853
GLUTATHIONE_METABOLISM	0.5047367	1.4905424	0.027455121
PENTOSE_AND_GLUCURONATE_INTERCONVERSIONS	0.58411217	1.4883057	0.03583815
LIPOIC_ACID_METABOLISM	0.95519745	1.46228	0.017889088
PHENYLALANINE_METABOLISM	0.48728165	1.4607399	0.049356222
FRUCTOSE_AND_MANNOSE_METABOLISM	0.4986104	1.4567932	0.042207792
CYSTEINE_AND_METHIONINE_METABOLISM	0.48944268	1.4549505	0.04821803
SESQUITERPENOID_AND_TRITERPENOID_BIOSYNTHESIS	0.8154585	1.446945	0.03587444
METABOLIC_PATHWAYS	0.35311216	1.2196815	0.002
BIOSYNTHESIS_OF_SECONDARY_METABOLITES	0.34965003	1.1944193	0.035

Amino acid metabolism mainly includes phenylalanine, cysteine, methionine, and glutathione metabolism. Thus, secondary metabolism is necessary for normal plant growth and development. A case in point, the phenylalanine metabolic pathway is an important secondary metabolic pathway in plants. Cysteine is an intermediate product of methionine metabolism, participating in the absorption of important sulfur elements by plants to form sulfur-containing vitamins and glutathione. Additionally, cysteine plays a role in the enhancement of sesquiterpene and triterpene biosynthesis. In turn, terpenoids, which are the most common category of plant secondary metabolites, play an important role in information exchange and interaction among plants, microorganisms, and insects; furthermore, they participate in the basic functions of plant photosynthesis and hormone metabolism. Altogether, our results suggest that *OsCIPK17* is involved in secondary metabolism and that it may play an important role in maintaining normal growth, development, and defense functions in rice.

### Differentially Expressed Genes Associations Based on Protein Interaction-Network Analysis

**FIGURE 7:**
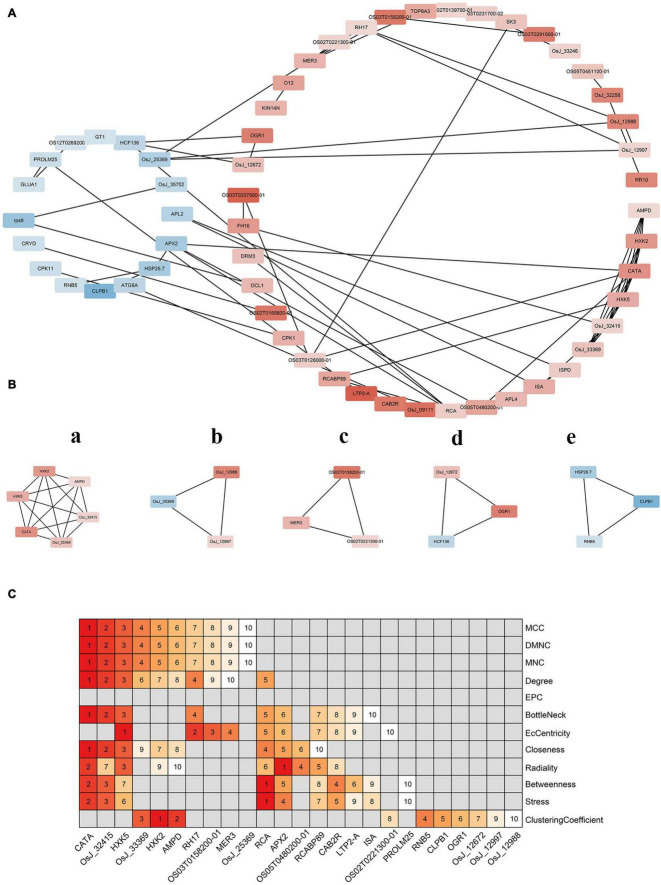
Protein interaction network analysis of differentially expressed genes. **(A)** Protein interaction regulatory network of differentially expressed genes. The up-regulated genes and down-regulated genes were divided into two groups. The deeper the red, the greater the expression; the darker the blue, the smaller the expression. **(B)** The important sub-modules of protein–protein interaction network are expressed in lowercase English letters according to the score. a has the highest score and e has the lowest score. **(C)** Key genes in protein–protein interaction network. On the right is the method of screening genes. The top 10 genes were sorted according to the score of each method. The higher the score, the higher the ranking.

Protein interaction networks are used to clarify associations among DEGs. As shown in Figure 7A, upregulated and downregulated proteins clustered into two classes. The number of upregulated proteins was higher than that of downregulated ones, indicating a more complex interaction relationship among them than that among downregulated proteins, implying that the interaction of differential proteins is not clearly concentrated in the upregulated or the downregulated proteins. Further, many upregulated and downregulated proteins were found to interact with each other. These results indicate that differential genes were not only co-expressed but also co-inhibited. Furthermore, some upregulated proteins, such as AMP deaminase (AMPD) and catalase isozyme A (CATA), showed significantly more interactions than others, suggesting that these proteins occupy key positions in the interaction network.

Further analysis of the protein network identified five more important submodules (Figure 7B). Among them, a is the most important sub-module, which is composed of 6 proteins and 15 pairs of interactions. Hexokinase 2 (HXK2) and hexokinase 5 (HXK5) belong to the hexokinase family, which catalyzes the phosphorylation of fructose and glucose, and may be involved in the phosphorylation of glucose for transport from plastids to the cytoplasm. AMPD is an AMPD that belongs to the superfamily of metal-dependent hydrolases and plays a key role in energy metabolism. CATA exists in almost all aerobic organisms and protects cells from hydrogen peroxide toxicity. OsJ_32415 and OSJ_33369 encode glucosyltransferase and pyruvate kinase, respectively. Thus, sub-module a may affect hydrogen peroxide isozyme by participating in glucose metabolism to protect rice from hydrogen peroxide toxicity. The other four modules were composed of three proteins and three pairs of interactions. OsJ_12988 in sub-module b encodes an RNA-binding protein. In turn, OsJ_12997 and OsJ_25369 encode an ATP-dependent RNA helicase, respectively, suggesting that sub-module b may be involved in RNA metabolism from translation initiation, ribosome formation, pre-mRNA splicing, and mRNA degradation. Interestingly, one of the two genes encoding helicase was upregulated but the other was downregulated, suggesting an antagonistic role in regulating RNA binding activity on protein expression. Sub-module c encodes two RNA helicases and one DNA helicase; its function may be similar to that of sub-module b. However, sub-module c has a distinct gene encoding DNA helicase that may provide the ability to interfere with meiosis to form gametes in rice. Lastly, sub-module d is composed of high chlorophyll fluorescence protein 136 (HCF136), opaque and growth retarding protein 1 (OGR1), and OsJ_12672. These last two proteins were upregulated. HCF136 encodes a component of PSII that is important for maintaining the stability of the photosynthetic system. OGR1 encodes a protein containing a polypeptide repeat sequence involved in multisite RNA editing and translates proteins in the electron transport chain associated with mitochondrial ATP production. Thus, OGR1 is seemingly necessary for RNA editing in the mitochondria. As for OsJ_12672, it encodes TIP41-like proteins that facilitate the transport of water and small neutral solutes across cell membranes and from vacuoles to the cytoplasm. Overall, sub-module d may affect photosynthesis and energy metabolism, and, ultimately, the transport of assimilates in rice cells. CLPB1 in sub-module e is a molecular chaperone that participates in the heat shock response. This protein may play a role in the redissolution of protein aggregates after heat shock. Both HSP26.7 and RNB5 encode heat shock proteins that belong to the small heat-shock protein 20 (HSP20) family, suggesting that the sub-module may be involved in rice stress responses.

Hub genes in the network were screened to identify the most important ones among DEGs. According to different algorithms, the top-10 key genes of each algorithm were screened out and a heat map was drawn according to their rankings. Figure 7C shows that CATA, Os_J32415, and HXK5 occupied very important positions. These results indicate that *OsCIPK17*-knockout might seriously affect the resistance of rice to hydrogen peroxide. In addition, RNA helicase 17 (RH17), helicase MER3 (MER3), ribulose diphosphate carboxylase/oxygenase activator (RCA), L-ascorbate peroxidase 2 (APX2), chlorophyll *a*/*b* binding protein (RCABP89), and chlorophyll *a*/*b* binding protein 2 (CAB2R) mainly encoded RNA, DNA helicase, and light-trapping protein, might also seemingly be affected, thus indicating that knockout of *OsCIPK17* would strongly impact RNA metabolism and photosynthesis in rice. The last category includes CLPB1 and RNB5, which can affect rice heat tolerance to a certain extent.

## Discussion

The CIPK protein family is widely distributed in plants and can regulate normal plant growth and development (Halfter et al., 2000; Yong et al., 2007; Chen et al., 2011a,^2013^; Li et al., 2014; Zhang et al., 2020). However, few CIPK proteins have been studied thoroughly. Further, due to differences among species and the number of family gene members in any given species, CIPK proteins can interact with CBL to produce different regulatory effects, whereby, CIPK proteins warrant further research. Studies have shown that many members of the CIPK family are stress-induced (Li et al., 2014; Peng et al., 2021), and they are known to be involved in the regulation of a wide array of BPs in plants, including seed germination, root growth, flowering, pollen tube growth, and stress responses (Xiang et al., 2007; Piao et al., 2010; Mahs et al., 2013; Xuan et al., 2019; Peng et al., 2021). Some CIPK members show variable expression patterns at different stages of plant growth (Yu et al., 2016), although there are few studies on plant CIPK17 in this respect, especially with regard to OsCIPK17 roles in rice.

In this study, first, the GFP fusion protein vector of *OsCIPK17* was constructed, and it was determined that the gene is likely located in the cytoplasm (Figure 2A). GUS staining confirmed that the gene was expressed in seedlings, root tips, rosette leaves, stems, flower buds, and pods; a finding that was further confirmed by quantitative analysis (Figure 2B). Then, grain weight and grain filling rate of superior grains were analyzed. Grain weight and G_max_ were lower in the mutant genotype than in the parent NIP (Figures 3A,B); furthermore, T_max_ was observed approximately 7 days later in the knockout mutant than in the parent line, indicating that the deletion of *OsCIPK17* was not beneficial for grain filling. In addition, we found that PSII energy use efficiency in mutant plants was significantly lower than that of the parent genotype at the middle grain-filling stage (Figure 3C). The same trend was observed for starch content (Figures 3D,E), suggesting that the reduced photosynthetic efficiency and starch content of the mutant may be the main physiological reason for the difference in grain filling at the later stage.

The main factors affecting rice yield were the number of panicles, grain number per panicle, seed-setting rate, and 1000-grain weight. Grain weight may be stably inherited. Therefore, understanding the molecular mechanism underlying the heredity of grain weight is a powerful means to increase yield. The involvement of the CIPK protein family in grain weight and grain filling has not been previously reported. Some known functions are related to seed germination, seedling and root growth, and flowering. For example, rice mutant *cipk31* showed a phenotype that was hypersensitive to ABA, salt, and mannitol during seed germination and seedling growth (Piao et al., 2010). In turn, *OsCIPK9* participates in NH_4_^+^-dependent root growth (Xuan et al., 2019), while an *OsCIPK3* mutation results in delayed flowering and late heading under conditions of long photoperiod (Peng et al., 2021). In this study, *OsCIPK17* knockout affected grain filling, leading to a decrease in rice grain weight (Figure 3 and Table 1).

In order to further clarify the molecular mechanism by which the *OsCIPK17* knockout mutation affected grain filling and led to grain weight reduction, we performed RNA-Seq on the superior grains of the parent and the mutant. Through PO enrichment analysis, we confirmed that DEGs were enriched in vegetative organs such as roots, stems, and leaves, as well as in reproductive organs including seeds, flowers, pistils, and even the component endosperm of rice (Figure 6A). GO enrichment analysis confirmed that the functions involving DEGs were mainly related to BPs and MFs (Figure 6B). MFs identified are mainly involved with the binding of nucleosides, nucleotides, and lipids, as well as enzyme activities, including hydrolases, transferases, and phosphorylase. GSEA enrichment analysis showed that the signaling pathways were mainly concentrated in starch and sucrose metabolism and photosynthesis (Figures 6C,D). In turn, functional enrichment in the KEGG database mainly focused on photosynthesis, photosynthate, and amino acid metabolism (Table 2). The differential genes are widely involved in the nutrition and reproductive organs of rice, and their functions are mainly related to photosynthesis, sucrose and starch synthesis, and amino acid metabolism. This suggests that the deletion of *OsCIPK17* may affect the synthesis and transport of photoassimilate, leading to a reduction in grain weight.

To clarify the connection between DEGs, a protein-interaction network was constructed (Figure 7A). There were more upregulated than downregulated proteins, and many upregulated proteins interacted with downregulated proteins. Furthermore, some upregulated proteins, such as AMPD and CATA, showed more interactions than other proteins. These results indicate that these proteins play a key role in the interaction network. Further analysis showed that the most important sub-modules were selected in the protein network (Figure 6B), whose functions are involved in protecting rice from damage caused by hydrogen peroxide and other reactive oxygen species, in participating in RNA metabolism and rice meiosis, and influencing the transport of assimilates and stress response by affecting photosynthesis and energy metabolism. In particular, sub-module d consisted of three proteins: HCF136, OGR1, and OsJ_12672. HCF136 encodes a PSII component. This protein has also been studied in other species. Thus, for example, mutations or deletions of the *HCF136* gene in *A. thaliana* and *Cyanobacteria* can lead to inactivation or loss of PSII function (Plücken et al., 2002; Komenda et al., 2008). This gene mutation has also been studied in maize, in which case it was found to block starch synthesis (Wu et al., 2018). In the experiments reported herein, downregulation of the *HCF136* gene in the mutant led to the obstruction of PSII function and affected starch synthesis, which may largely explain the reduction observed in grain weight. OGR1 encodes a protein containing polypeptide repeats and participates in mitochondrial multipoint RNA-editing. Previous studies showed that OGR1 is located in the mitochondria of rice. Mutant rice shows a multidirectional effect, slow growth, and late flowering, resulting in a dwarf, sterile phenotype (Kim et al., 2010). The *cipk17*-mutant rice phenotype with reduced maximum filling rate and grain weight may be related to the upregulated expression of the OGR1 gene. In turn, OsJ_12672 encodes a TIP41-like protein that promotes the transport of water and small neutral solutes across the cell membrane and from the vacuole to the cytoplasm. Previous studies have shown that deletion of TIP41 can increase yeast resistance to mold (Jacinto et al., 2001). It can also participate in the ABA signaling pathway in *A. thaliana* and regulate the developmental process in this species (Punzo et al., 2018). Here, the upregulation of this gene may largely explain the observed sustainment of grain filling rate of superior grains in the *cipk17* rice mutant.

## Conclusion

**FIGURE 8:**
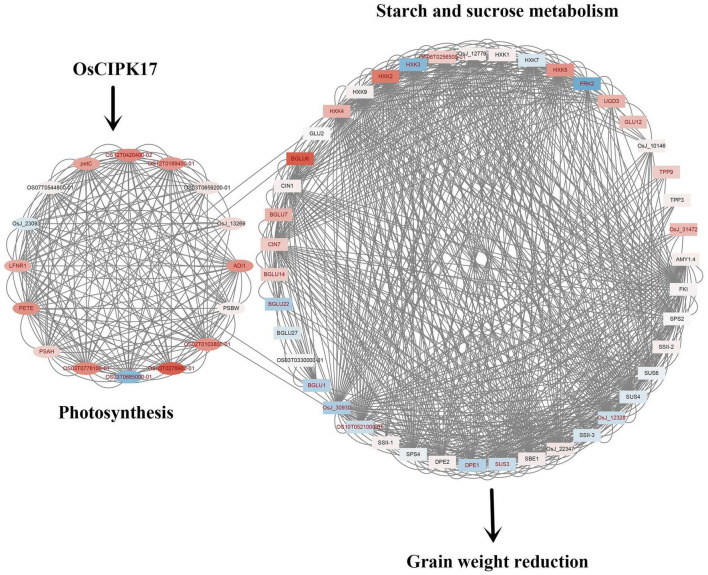
Mechanism diagram of OsCIPK17 affecting rice grain weight. OsCIPK17 can affect photosynthesis indirectly or directly. The deficiency of photosynthesis results in impaired metabolism of starch and sucrose, resulting in decreased grain weight. The up-regulated genes and down-regulated genes were mapped with different colors. The deeper the red, the higher the expression; the darker the blue, the smaller the expression. The names of genes with significant differences in expression are indicated in red. The proteins linked by lines were predicted to have interactions.

In this study, we found that *OsCIPK17* was localized in the cytoplasm. Gus staining of heterologous tissues showed that the *OsCIPK17* gene was expressed in seedlings, in the root maturation zone, in rosette leaves, stems, and flower buds but not in the seed coat or the embryo. Upon *OsCIPK17* knock out mutation, the maximum filling rate of superior grains decreased in the *cipk17* mutant, the grain filling process shifted backward, the duration of grain filling became shorter and, consequently, final grain weight decreased. It is likely that *OsCIPK17* gene knockout affected the expression of genes related to starch and sucrose (Figure 8), while concomitantly inhibiting enzyme activity or hindering protein synthesis and photosynthetic efficiency, finally affecting starch and sucrose transport and accumulation, consequently reducing grain weight.

## Data Availability Statement

The raw sequence data have been deposited in the Genome Sequence Archive in National Genomics Data Center, China National Center Bioinformation/Beijing Institute of Genomics, Chinese Academy of Sciences (GSA: CRA005385) that are publicly accessible at https://ngdc.cncb.ac.cn/gsa.

## Author Contributions

CG, XZ, SL, JX, RZ, JL, and YaC performed the material preparation, data collection, and analysis. CG and YuC wrote the first draft of the manuscript and all authors commented on previous versions of the manuscript. YuC critically revised the manuscript. All authors have contributed to the study conception and design, read and approved the manuscript.

## Conflict of Interest

The authors declare that the research was conducted in the absence of any commercial or financial relationships that could be construed as a potential conflict of interest.

## Publisher’s Note

All claims expressed in this article are solely those of the authors and do not necessarily represent those of their affiliated organizations, or those of the publisher, the editors and the reviewers. Any product that may be evaluated in this article, or claim that may be made by its manufacturer, is not guaranteed or endorsed by the publisher.
