# Supplementary material for: Functional Analysis of OsCIPK17 in Rice Grain Filling
Source: Front Plant Sci. 2022 Jan 25;12:808312. doi: 10.3389/fpls.2021.808312 (PMC8821165; doi:10.3389/fpls.2021.808312)
Supplement: Supplementary file 1 [file Table_1.DOCX]

**Supplementary Table 1 Primers used for identification and vector construction**

| Direction | Primer sequence | Purpose |
| --- | --- | --- |
| Forward | GCCAATCCGTCTTCCATTATATTTCTC | Identification |
| Reverse | GGAGGCGAACCACGCAATTT | (clone) |
| Forward | ATGGGGAGGACGCTCGGGGA | Identification |
| Reverse | GAGCCTAGTCTACGTGCGGCTGCG | (primer amplification at the target) |
| Forward | GACGAGCTGTACAAGGGATCCATGGTGAAGGGAGGGAGGGA | eGFP vector construction |
| Reverse | CTGCAGGTCGACTCTAGATTACAAAGCGAACAGCGGC |  |
| Forward | CACCGCGAAGGCTGACGATAGACCATG | GUS vector construction |
| Reverse | CGCGTACTTCACCTTGCCGAAG |  |

**Supplementary Table 2 Primers used for qRT-PCR**

| Gene ID | Direction | Primer sequence | Length (bp) |
| --- | --- | --- | --- |
| LOC_Os02g09140 | forward | CTTCATTACATCTGGTGGAGACC | 147 |
|  | reverse | CCTTGATTTTCAGCATCCTCGTC |  |
| LOC_ Os04g58700 | forward | AAGTACGGGGAGGAGATCAAC | 131 |
|  | reverse | TCGGCGATGTACACCTTCTTG |  |
| LOC_Os03g61890 | forward | AGAGGGTGAAAAGGTGCTTCC | 143 |
|  | reverse | TCCGAAAACGCAGGCTAAAG |  |
| LOC_Os11g34370 | forward | CGACTAGGATTGCAAGGGATTG | 116 |
|  | reverse | TTGTCTAGAACCTCGTCTTCGG |  |
| LOC_Os09g39560 | forward | GAATGAAAAGCGAGCAAGCG | 120 |
|  | reverse | AAAAGGCAGTTCCACGTAGG |  |
| LOC_Os08g43170 | forward | ATGGCCAGCGAATTCTCATG | 128 |
|  | reverse | TGTCGTCACATCAAGCACAG |  |
| LOC_Os04g45600 | forward | TACACGCTTCGTTTGATCGG | 110 |
|  | reverse | CCCATGCTGAAATTGAGGACTC |  |
| LOC_Os01g41710 | forward | CATCACCATGCGCAAGTCG | 130 |
|  | reverse | GAACTCGCCGGTCAGGTAG |  |
| LOC_Os02g49070 | forward | TGATGATATCATGGGGGAAGCG | 134 |
|  | reverse | TCACCAGGGCATTGTCTTTG |  |
| LOC_Os10g24090 | forward | GAATCCCGGGGTTCCTCTTC | 133 |
|  | reverse | GCGAGCAGCTTCCTAAAGTG |  |
| LOC_Os04g51460 | forward | CGGTACCGCAACTTCACC | 132 |
|  | reverse | GCAGTAGTCGTACACCATGTAG |  |
| LOC_Os03g03660 | forward | TACCACAATCGGTTCCCAAGG | 100 |
|  | reverse | TGTTCACCTCAGGCCAAGTATC |  |
| LOC_Os10g41250 | forward | ACCTCCGAATTCAGCCGTAG | 106 |
|  | reverse | CCGATTTGGCAACTGGGTAG |  |
| LOC_Os07g47250 | forward | TACAGGCATTCGTTGGTGTG | 142 |
|  | reverse | GCGTTAGGCATGTTAGGATAGC |  |
| Actin-1 | forward | CTTCATAGGAATGGAAGCTGCGGGTA | 197 |
| (LOC_Os03g50885) | reverse | CGACCA CCTTGATCTTCATGCTGCTA |  |
